# Weight, insulin resistance, blood lipids, and diet quality changes associated with ketogenic and ultra low-fat dietary patterns: a secondary analysis of the DIETFITS randomized clinical trial

**DOI:** 10.3389/fnut.2023.1220020

**Published:** 2023-07-12

**Authors:** Lucia Aronica, Matthew J. Landry, Joseph Rigdon, Christopher D. Gardner

**Affiliations:** ^1^Stanford Prevention Research Center, Stanford University School of Medicine, Stanford, CA, United States; ^2^Department of Biostatistics and Data Science, Wake Forest University School of Medicine, Quantitative Sciences Unit, Stanford, CA, United States

**Keywords:** ketogenic diet, ultra low-fat diet, low carbohydrate, low fat, weight loss, triglycerides/HDL ratio, insulin resistance, refined grains

## Abstract

**Background:**

The DIETFITS trial reported no significant difference in 12-month weight loss between a healthy low-fat and healthy low-carbohydrate diet. Participants were instructed to restrict fat or carbohydrates to levels consistent with a ketogenic or ultra low-fat diet for 2  months and to subsequently increase intakes until they achieved a comfortable maintenance level.

**Objective:**

To compare 3- and 12-month changes in body weight and cardiometabolic risk factors between a subsample of participants who reported 3-month fat or carbohydrates intakes consistent with either a ketogenic-like diet (KLD) or ultra low-fat diet (ULF).

**Design:**

3-month and 12-month weight and risk factor outcomes were compared between KLD (*n* = 18) and ULF (*n* = 21) sub-groups of DIETFITS participants (selected from *n* = 609, healthy overweight/obese, aged 18–50  years).

**Results:**

Less than 10% of DIETFITS participants met KLD or ULF criteria at 3-months. Both groups achieved similar weight loss and insulin resistance improvements at 3-months and maintained them at 12- months. Significant differences at 3-months included a transient ~12% increase in LDL cholesterol (LDL-C) for KLD with a concomitant greater reduction in log(TG/HDL), a measure of LDL-C’s atherogenic potential. The latter was maintained at 12-months, despite substantial diet recidivism for both groups, whereas LDL-C levels were similar for ULF at baseline and 12-months. KLD participants achieved and maintained the greatest reductions in added sugars and refined grains at 3- months and 12-months, whereas ULF participants reported a 50% increase in refined grains intake from baseline to 12-months.

**Conclusion:**

Among the ~10% of study participants that achieved the most extreme restriction of dietary fat vs. carbohydrate after 3  months, weight loss and improvement in insulin sensitivity were substantial and similar between groups. At 12  months, after considerable dietary recidivism, the few significant differences in diet quality and blood lipid parameters tended to favor KLD over ULF.

## 1. Introduction

The consumption of added sugars and refined carbohydrates has significantly grown in the past five decades with a concomitant increase in the global rates of obesity, diabetes and cardiovascular disease (CVD) (1, 2). While a substantial reduction in added sugars and refined carbohydrate intake has become a priority in current public health guidelines (3), there is still little consensus on the optimal ratios of carbohydrates and fats for promoting weight loss and optimizing CVD risk factors. Multiple diets promoting reduction of added sugar and refined grain consumption with varying ranges of carbohydrate and fat content have been associated with significant improvements in body weight and cardiovascular disease (CVD) risk factors (4). At one extreme is the ketogenic diet, which restricts net carbohydrates to ≤20–50 g per day (5–13), and at the opposite end of the spectrum are ultra low-fat diets like the Ornish and Pritikin diets, which recommends a drastic reduction of fat to <10% of total daily calories (14–16).

Several randomized clinical trials (RCTs) have compared weight loss and chronic disease factors on low-carbohydrate (LC) and low-fat diets (LF), with a preponderance of studies reporting greater benefits for LC at 6-months but not at 12-months (17–20). However, these studies displayed a high variability in the definitions of “low-carb” and “low-fat” and in the reporting of adherence to these two dietary approaches (21, 22). We recently reported that there was no significant difference in 12-month weight loss among 609 healthy subjects with overweight/obesity assigned to a Healthy Low Carbohydrate diet (HLC) or Healthy Low Fat diet (HLF) (23). However, both diets minimized added sugar and refined grains and, hence, reduced overall carbohydrate consumption from baseline (23). In addition, with such a large study population, variability in both adherence and weight loss success was substantial. Following the main publication of our findings, we received many queries regarding the outcomes for the subset of participants in the study that had been most adherent and achieved the greatest dietary changes from their baseline diets for both intervention diets. This analysis is a response to those queries.

In order to explore the potential health impacts of diets with greater differentiation in relative carbohydrate and fat content, in this secondary analysis of the DIETFITS study we selected the participants from the HLC group who reported achieving the greatest carbohydrate restriction - a dietary pattern that resembled a ketogenic diet (ketogenic-like diet, KLD), and the participants from the HLF group who reported achieving the greatest fat restriction - a dietary pattern that resembled an ultra low-fat diet (ultra low-fat, ULF). The 3-month intermediate time point was used for selecting these subsets because this was when participant enthusiasm and engagement was assessed to be highest; at the end of the 12-month protocol very few individuals from the original n = 609 DIETFITS participants reported dietary patterns that resembled KLD or ULF. Changes in weight and chronic disease risk factors were contrasted between these two selected subgroups at the 3-month time point, and then longer-term dietary pattern adherence was examined at 12-months, along with weight and risk factor comparisons.

## 2. Subjects and methods

### 2.1. Design and participants

The original trial, Diet Intervention Examining The Factors Interacting with Treatment Success (DIETFITS), was a single-site, parallel-group, randomized trial of 609 men (*n* = 263) and women (n = 346) with overweight or obesity conducted at the Stanford Prevention Research Center from January 2013 to May 2016 and designed to whether baseline genetic or cardiometabolic factors would explain differential weight loss for those assigned to either a HLC or a HLF diet (23).The detailed primary study protocol has been reported elsewhere (24). Participants were generally healthy women and men, aged 18–50 years, with body mass index (BMI) 28–40 kg/m^2^. The dietary protocol consisted of two phases, *Limbo* and *Titrate*, whose goal was to help participants achieve the lowest intake of fat or carbohydrates they could realistically maintain beyond the end of the trial. During the first eight weeks of *Limbo* phase, participants were instructed to cut back on fat or carbohydrate intake progressively until they achieved a daily intake of no more than 20 g of carbohydrate (HLC) or fat (HLF), which is consistent with a ketogenic or ultra low-fat dietary pattern, respectively. During the *Titrate* phase, participants were instructed to increase their fat or carbohydrate intake slowly, by 5–15 g each week, until they achieved a comfortable maintenance level. In this phase participants were instructed to strive for the lowest intake of fat or carbohydrates they could realistically maintain for the 12-month intervention period, and even beyond the end of the trial should they experience positive benefits from their diet assignment. Emphasis on diet *Quality* was a common feature of both intervention arms. All subjects were instructed to (1) maximize vegetable intake; (2) minimize added sugars, refined grains, and trans fats; and (3) focus on minimally-processed whole foods, prepared at home when possible.

### 2.2. Dietary assessment

Dietary intake was recorded using unannounced 24-h multiple-pass recall interviews. Diet recalls were collected using Nutrition Data System for Research (NDS-R, University of Minnesota), a computer-based dietary analysis program designed for the collection and analyses of 24-h dietary recalls. Nutrient profiles are compiled using data provided by the NDSR software and sourced from the NCC Food and Nutrient Database (25). Data from 3 dietary recalls for each time point (baseline, 3-, 12-months), 2 on weekdays and 1 on a weekend day, were averaged and used to determine overall dietary intake for each time point. Six-month data were available, but not included in this analysis.

### 2.3. Anthropometric and laboratory measures

Anthropometric measures and blood samples were captured at baseline, 3-, and 12- months. Body weight was recorded without shoes to the nearest 0.1 kg using a calibrated Scale-tronix clinical scale. Height was measured to the nearest 0.1 cm using a Seca wall-mounted stadiometer. All measurements were taken by a nurse at the Stanford Clinical & Translational Research Unit (CTRU) at each time point. All clinic visits started between 7:00 and 9:30 am, with participants in a fasted state for at least 10–12 h. Blood samples were taken at baseline, 3, 6 and 12 months via venipuncture by trained nurses or phlebotomists. Blood was collected into purple top EDTA vacutainer tubes. Samples were processed, aliquoted, and frozen directly by the CTRU lab after being drawn. Samples were stored in a − 80° freezer until the time of processing for analysis. Lipids were assessed at all four times points (i.e., baseline, 3, 6, and 12 months) from a fasting blood sample. Plasma triglycerides, total- and HDL-cholesterol were measured by enzymatic endpoint analysis on a clinical chemistry analyzer (Liasys 330). LDL-cholesterol was calculated using the Friedewald equation. Triglyceride and cholesterol measurements are standardized through the CDC-NHLBI lipid standardization program. Insulin levels were assessed by radioimmunoassay by the Core Laboratory for Clinical Studies Washington University School of Medicine, St. Louis, Missouri. Glucose levels were analyzed using a Beckman Glucose Analyzer II (BGA II) by electrochemical technique. Insulin resistance status was determined by calculating the Homeostasis Model Assessment of Insulin Resistance (HOMA-IR) as described previously (26). 6-month data were available, but not included in this analysis.

### 2.4. Diet group criteria and outcome analysis

KLD and ULF subjects were selected at 3-months, as this was the timepoint with the highest reported restriction of carbohydrates or fat, and the most complete weight and cardiometabolic data (i.e., least drop-out). Out of 549 subjects with intake data at 3-months, we conservatively excluded those who reported <1,200 kcal per day due to underreporting concerns (*n* = 128/549 subjects excluded, 23%). For KLD, the threshold of net carbohydrates (carbohydrates minus fiber) was set at <30 g per day, based on the recommended range of 20-50 g used in previous studies (27). This resulted in the inclusion of 18/205 subjects in KLD from the HLC group. For ULF the cutoff of fat intake was initially defined as <10% of daily calories from fat based on original recommendations of the Ornish diet (28). However, since only 5 of 216 subjects met the <10% fat cutoff, this was increased to 15% (about 20 g/day) that resulted in the identification of 21 ULF subjects, a number reasonably comparable to the KLD group.

### 2.5. Statistics

The primary aim of this study was to test whether changes in baseline to 3-month outcomes [weight, HDL-C, triglycerides, log(TG/HDL-C), LDL-C, and HOMA-IR] were different among the two study population subgroups; KLD and ULF. Baseline demographic, anthropometric and cardiometabolic variables data are presented using basic descriptive data. Patterns of nutrition intake at baseline, 3- and 12-months were also summarized descriptively using means and standard errors by diet and timepoint.

Linear mixed effects models (29) with fixed effects for diet, time (baseline, 3-, or 12-months), and all diet by time interactions, and a random intercept for participant were used to test all primary study hypotheses to account for the correlated nature of within participant changes while using all available data. The models allowed estimation of within-diet baseline to 3-month changes and also comparisons (using two-sided Wald tests) between diets of these 3- and 12-month changes. No adjustments for multiple testing were made given that this was a hypothesis generating secondary analysis of a subset of participants from a large, randomized trial. All statistical tests were two-tailed with type 1 error assumed to be 0.05. All analyses were conducted using R version 4.1.2 (30).

## 3. Results

### 3.1. Baseline characteristics of the study population

The KLD (*n* = 18) and ULF (*n* = 21) groups included participants exclusively from the HLC and HLF arms, respectively. There were no significant between-group differences in baseline demographic or anthropometric data and laboratory measurements (Table 1). Baseline daily intake of total calories, fats, protein and added sugars and refined grains was also similar between groups (Table 2**)**. The ULF group reported a marginally significantly greater baseline daily intake of total carbohydrates compared to the KLD group (KLD: 250.4 ± 18.3; ULF: 292.4 ± 17.7 grams; *p* = 0.047). However, baseline daily intake of net carbohydrates (total carbohydrates minus fiber) was similar between the two groups.

**Table 1:** Baseline demographics and cardiometabolic variables for KLD and OLD.

	KLD	ULF	*p*-value^a^
	*n*=18	*n*=21	
Diet			
Healthy Low Carb	18 (100.0%)	0 (0.0%)	<0.0001
Healthy Low Fat	0 (0.0%)	21 (100.0%)	
Sex			
Female	9 (50.0%)	7 (33.3%)	0.60
Male	9 (50.0%)	14 (66.7%)	
Age (years)	42.0 (±6.8)	41.2 (±5.6)	0.50
Weight (kg)	103.8 (±14.3)	102.8 (±15.6)	0.32
Race/ethnicity			
White	16 (88.9%)	14 (66.7%)	0.26
Hispanic	2 (11.1%)	3 (14.3%)	
Asian	0 (0.0%)	3 (14.3%)	
Other	0 (0.0%)	1 (4.8%)	
HDL	47.4 (±10.7)	47.0 (±8.8)	0.35
LDL	121.0 (±33.1)	109.4 (±27.7)	0.53
Triglycerides	219.1 (±29.1)	137.0 (±67.6)	0.22
Log(TG/HDL ratio)	1.2 (±0.9)	1.0 (±0.4)	0.44
HOMA-IR	4.7 (±2.5)	4.3 (±3.6)	0.88
DXA percent fat	36.5 (±6.5)	34.1 (±5.8)	0.55
Missing	6 (33.3%)	9 (42.9%)	
BMI (kg/m^2^)	35.0 (±2.7)	33.3 (±3.2)	0.065

a Wilcoxon rank-sum for continuous variables, e.g., age, and Fisher’s exact test for categorical variables, e.g. race.

**Table 2:** Nutrition variables for KLD and ULF at baseline, 3 months, and 12 months.

	KLD	ULF	*p*-value^a^
	*n*=18	*n*=21	
Calories (kcal/day)			
Baseline	2265 (±133)^b^	2392 (±136)	0.42
3 Months	1482 (±63)	1543 (±45)	0.70
12 Months	1665 (±118)	1938 (±145)	0.13
Carbohydrates (g/day)			
Baseline	250 (±18)	292 (±18)	0.05
3 Months	38 (±4)	264 (±8)	<0.0001
12 Months	96 (±14)	264 (±20)	<0.0001
Net carb (g/day)			
Baseline	226 (±17)	265 (±17)	0.05
3 Months	16 (±7)	227.3 (±7)	<0.0001
12 Months	77 (±12)	236 (±19)	<0.0001
Fat (g/day)			
Baseline	97 (±7)	86.2 (±6.5)	0.23
3 Months	100 (±6.0)	20 (±1.1)	<0.0001
12 Months	90.3 (±9.3)	51.9 (±5.8)	<0.0001
Protein (g/day)			
Baseline	91 (±5)	100 (±4)	0.33
3 Months	110 (±7)	84 (±6)	0.01
12 Months	109 (±11)	98 (±6)	0.28
Added sugars (g/day)			
Baseline	54 (±8)	45 (±7)	0.25
3 Months	3 (±1)	23 (±4)	0.01
12 Months	10 (±3.4)	32 (±5)	0.01
Refined grains (g/day)			
Baseline	93 (±18.1)	114 (±24)	0.47
3 Months	3 (±1)	81 (±22)	<0.01
12 Months	41(±13)	166 (±30)	<0.001
Fat (%)			
Baseline	37.2 (±1.1)	31.3 (±1.3)	<0.01
3 Months	58.6 (±1.9)	11.1 (±0.5)	<0.001
12 Months	46.9 (±2.8)	22.4 (±1.3)	<0.001
Carbohydrates (%)			
Baseline	43.5 (±1.9)	48.4 (±1.7)	0.07
3 Months	9.8 (±1.3)	66.4 (±1.8)	<0.001
12 Months	22.7 (±3.2)	53.4 (±1.8	<0.001
Protein (%)			
Baseline	16.7 (±0.9)	17.2 (±0.7)	0.76
3 Months	30.9 (±1.4)	21.0 (±1.4)	<0.001
12 Months	27.3 (±2.1)	21.1 (±1.2)	<0.01
Sugar (%calories)			
Baseline	22.6 (±2.9)	18.0 (±2.4)	0.21
3 Months	2.0 (±0.5)	16.2 (±3.4)	<0.001
12 Months	6.0 (±2.1)	17.6 (±2.7)	<0.01
Total sugars (g)			
Baseline	93.6 (±8.6)	105.6 (±11.1)	0.73
3 Months	16.5 (±2.3)	94.7 (±6.0)	<0.001
12 Months	35.2 (±5.1)	95.3 (±11.1)	<0.001

a *p*-values for null hypothesis that nutrition variables are equivalent between diets at a given timepoint; from a linear mixed effects model including fixed effects for time, diet, and time*diet interaction, and a random effect for study participant.

b Mean (±standard error of mean).

### 3.2. Intake of macronutrient, added sugars, and refined carbohydrates

**Figure 1:**
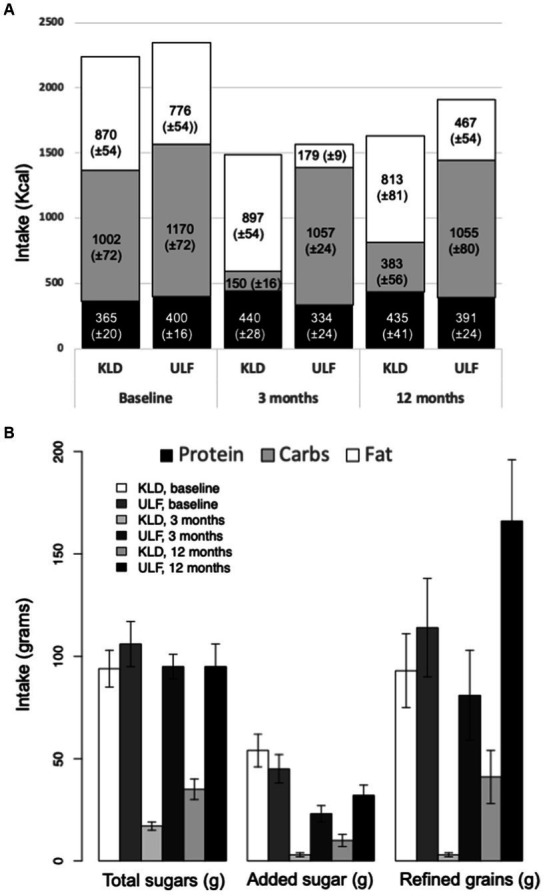
Macronutrient, added sugar, and refined grains intake for KLD and ULF. **(A)**: Mean intake (Kcal/day; ± standard error of mean) of protein (black), carbohydrates (gray), and fat (white) for KLD and ULF at baseline, 3 months, and 12 months. **(B)**: Mean intake (grams/day; ± standard error of mean) of total sugar, added sugars, and refined grains for KLD and ULF at baseline, 3 months, and 12 months. *p*-values for null hypothesis that nutrition variables are equivalent between diets at a given timepoint; from a linear mixed effects model including fixed effects for time, diet, and time*diet interaction, and a random effect for study participant.

All groups reported similar reductions in caloric intake at 3-months relative to baseline. As expected, and by design, there were significant between group differences in the intake of macronutrients (Table 2 and Figures 1A,B). Although all DIETFITS participants were instructed to minimize added sugars and refined carbohydrates, their reduction was significantly greater for KLD than ULF at 3-months with an average difference in daily intake of ~20 g of added sugars and ~ 80 g of refined grains between KLD vs. ULF. As presented in Table 2, net carbohydrate intake (g/day) was significantly lower for KLD vs. ULF (KLD: 15.5 ± 7.2; ULF: 227.3 ± 6.5; *p* = <0.001), whereas fat intake was significantly lower for ULF vs. KLD (KLD: 99.7 ± 6.0; ULF: 19.9 ± 1.1; *p* = <0.001). Protein intake (g/day) was significantly higher for KLD compared to ULF (KLD = 110.1 ± 7.1 g; ULF = 83.5 ± 5.9 g; *p* = <0.001).

The pattern of statistical differences between the two groups persisted at 12-months, although the reported intakes of carbohydrates and fat increased for KLD and ULF, respectively. For KLD, the reported net carbohydrate intakes increased ~60 g/day reaching ~80 g/day, whereas fat intakes remained relatively stable around 90 g/day. For ULF, the reported fat intakes increased ~30 g/day reaching 50 g/day, whereas carbohydrate intakes remained relatively stable at around 230 g/day. At 12-month the reported intakes of refined grains increased for both KLD and ULF from 3-months. However, they remained >50% lower than at baseline for KLD, whereas they were almost 50% higher than at baseline for ULF. The reported 12-month added sugars intake was higher than at 3-months but lower than at baseline for both groups.

### 3.3. Changes in weight, blood lipids, and insulin resistance

At 3-months, weight loss was similar between KLD and ULF (Table 3). LDL-C decreased by ~3% for ULF, whereas it increased by ~12% for KLD with a significant between-group difference [KLD: 14.9 mg/dL (3.4, 26.4); ULF: −2.9 mg/dL (−13.8, 8.0); *p* = 0.03]. On the other hand, compared to ULF, KLD resulted in a significantly greater reduction in the log(TG/HDL-C) also known as atherogenic index of plasma (AIP), a measure of the atherogenic potential of an individual’s LDL profile [KLD: −0.53 (−0.77, −0.28); ULF: −0.13 (−0.36, 0.11); *p* = 0.02]. Both KLD and ULF resulted in a similar ~30% reduction in insulin resistance (HOMA-IR) from baseline that persisted at 12-months with no significant between-group difference.

**Table 3:** Baseline to 3-month changes in clinical variables for KLD and ULF.

	KLD	ULF	*p*-value^a^
Weight (kg)^a^	-9.8 (-12.8, -6.8)	-8.7 (-11.6, -5.8)	0.59
HDL (mg/dl)	1.7 (-1.8, 5.1)	-3.0 (-6.4, 0.3)	0.05
LDL (mg/dl)	14.9 (3.4, 26.4)	-2.9 (-13.8, 7.9)	0.03
Trig (mg/dl)	-108.6 (-184.8, -32.4)	-25.1 (-97.8, 47.6)	0.12
Log (TG/HDL ratio)	-0.53 (-0.77, -0.28)	-0.13 (-0.36, 0.11)	0.02
HOMA-IR	-1.4 (-2.3, -0.4)	-1.4 (-2.3, -0.4)	0.98
Sugar (%calories)	-20.6 (-25.9, -15.2)	-1.84 (-6.8, 3.1)	<0.001
Total sugars (g)	-77.1 (-97.1, -57.1)	-11.0 (-29.5, 7.5)	<0.001

a *p*-values, estimates, and 95% confidence intervals from a linear mixed effects model including fixed effects for time, diet, and time*diet interaction, and a random effect for study participant.

At 12-months, when substantial dietary recidivism was reported by both the KLD and ULF groups, LDL-C and weight loss were similar for KLD and ULF, whereas KLD maintained significantly greater improvements in log(TG/HDL) compared to ULF [KLD: −0.62 (−0.87, −0.37); ULF: −0.09 (−0.32, 0.15); *p* = 0.003 (Table 4)]. Overall, at 12-months both KLD and ULF lost ~10 kg of body weight and experienced a ~ 30% reduction in insulin resistance, whereas TG and HDL changed similarly and only modestly in the ULF groups at 12-months.

**Table 4:** Baseline to 12-month changes in clinical variables for KLD and ULF.

	KLD	ULF	*p*-value
Weight (kg)^a^	-9.9 (-12.9, -6.9)	-10.5 (-13.3, -7.6)	0.78
HDL (mg/dl)	4.9 (1.3, 8.5)	0.1 (-3.3, 3.5)	0.05
LDL (mg/dl)	5.0 (-6.7, 16.7)	-3.3 (-14.1, 7.6)	0.31
Trig (mg/dl)	-113.7 (-191.2, -36.2)	-13.0 (-85.7, 59.7)	0.06
Log TG/HDL ratio	-0.62 (-0.87, -0.37)	-0.09 (-0.32, 0.15)	<0.01
HOMA-IR	-1.3 (-2.3, -0.30)	-1.6 (-2.5, -0.6)	0.67
Sugar (%calories)	-16.6 (-22.2, -11.1)	-1.1 (-6.2, 4.0)	<0.001
Total sugars (g)	-57.9 (-78.6, -37.1)	-10.8 (-29.9, 8.4)	0.001

a *p*-values, estimates, and 95% confidence intervals from a linear mixed effects model including fixed effects for time, diet, and time*diet interaction, and a random effect for study participant.

## 4. Discussion

In this secondary analysis of the DIETFITS study we examined 3-month and 12-month changes in weight loss and CVD risk factors among the <10% of participants who reported consuming a very-low carbohydrate ketogenic-like diet (KLD) or an ultra low-fat diet (ULF) at 3-months. Compared to ULF, KLD resulted in a transient but significantly greater increase in LDL-C at 3-months with a concomitant significantly greater reduction in the log(TG/HDL). At 12-months, LDL-C was similar for KLD and ULF, whereas KLD maintained significantly greater improvements in log(TG/HDL) compared to ULF. In terms of diet quality, refined grain intake was reduced significantly more for KLD than ULF at both 3-months. At 12-months the consumption of refined grains remained less than 50% of baseline levels for KLD, whereas it increased almost 50% from baseline for ULF, possibly due to compensatory mechanisms for the decreased intake of calories from fat. Both groups substantially reduced added sugars, although these reductions were significantly greater for KLD compared to ULF at both 3-months and 12-months; KLD and ULF reported consuming ~80% and ~ 30% less added sugar than at baseline, respectively.

Our analysis provides a snapshot of the DIETFITS participants assigned to HLC or HLF that most successfully restricted dietary carbohydrates or fat, respectively, at 3-months. Participants were instructed to consume <20 g of carbohydrates or fat for the HLC or HLF, respectively, during the first 2 months of *Limbo* phase, and to subsequently increase intakes until they achieved their lowest level of intake that they could realistically maintain in the long term. Following these instructions, few subjects assigned to either diet arm achieved and maintained at 3-months macronutrient intakes approximating those initial targets: <30 g/day of carbohydrates for the 18 subjects in KLD out of 205 assigned to HLC, and < 15% of fat, equivalent to about 20 g/day, for the 21 subject in ULF out of 216 assigned to HLF. These dietary patterns underscore notable parallels: KLD aligns with the principles of the Atkins induction diet, while ULF shares resemblances with well-known ultra low-fat regimens like the Ornish and Pritikin diets. Even these subjects who most successfully restricted dietary carbohydrates or fat at 3-months reported substantial overall recidivism toward baseline intake values of carbohydrates or fat by 12-months. Nevertheless, ULF maintained an average fat intake of ~50 g/day at 12-months, and KLD maintained an average intake of ~80 g/day of net carbohydrates. Compared to the larger DIETFITS population (23), at 12-months both KLD and ULF lost twice as much weight (~ −10 kg vs. ~ −5 kg) and experienced a two times greater improvement in insulin resistance (30% vs. 15%).

Our finding that a very low-fat diet may lead to a compensatory increase in refined grain intake is in line with what was reported by the Women’s Health Initiative (WHI) Dietary Modification Trial, which showed that women who consumed a low-fat diet (<20% daily energy intake) increased intake of refined grains (+0.3 servings/d) (31). This suggests that, while a low fat diet has the potential to be cardioprotective if the total sugar intake is also kept low, more often there is a compensatory increase in the consumption of refined carbohydrates and added sugars. It is estimated that dietary carbohydrate intake among US adults make up 50% of our total energy intake with over 40% of carbohydrates being of low-quality from refined grains, added sugars in foods and beverages, fruit juice, and potatoes (32).

Our data add to previous evidence indicating that very low-carb ketogenic diets can lead to a triad of higher LDL-C, higher HDL-C and lower TGs (5–10, 12, 13, 33–45). This triad is thought to reflect a shift toward an overall less atherogenic LDL profile (46, 47) — from TG-enriched small dense LDL particles (LDL-P, pattern B) to cholesterol-enriched large buoyant LDL-P (pattern A) (5, 11, 42, 48, 49). Specifically, KLD induced a ~ 12% transient increase in LDL-C at 3-months with a concomitant greater decrease in log(TG/HDL), which is a marker of increased LDL-P size and an overall less atherogenic LDL profile (50). At 12-months, KLD maintained this greater improvement in log (TG/HDL), whereas LDL-C was similar for KLD and ULF.

This study has several strengths including a large parent trial (*n* = 609), comprehensive diet assessment, comprehensive set of cardiometabolic risk factors analyzed, and high macronutrient differentiation at 3-months in presence of similar caloric intake and reduction of added sugars and refined grain intake. Our analysis also has a number of important limitations. First, this was a post-hoc analysis that tested hypotheses that were not planned in the parent trial protocol. Most importantly, only a small number of subjects met the criteria for inclusion compared to the broader parent trial, which impairs the significance and generalizability of our findings. In addition, dietary intake data were self-reported even if collected in multiple pass recalls. Therefore, all the reported statistically significant associations or lack thereof must be interpreted with caution. For example, despite non-statistically different baseline values in triglycerides, the physiological differences could still contribute to the observation that triglycerides and the atherogenic index improved in KLD. Finally, it is important to mention the difficulty in discerning the impact of diet versus weight loss on clinical outcomes and that long-term studies on ketogenic diets are limited.

Clinicians can take away a few practical insights from this exploratory analysis. First, patients who successfully establish a very low “anchor” of carbohydrate or fat intake in the initial phase of a low-carb or low-fat intervention may achieve lower maintenance intakes and better outcomes than those who began with a higher anchor. Anchoring is an unconscious process whereby initial exposure to a number serves as a reference point or “anchor” thus influencing subsequent judgments (51–54). Both KLD and ULF participants achieved greater long-term restrictions of carbohydrates and fats, respectively, and greater weight loss and insulin improvements than the overall DIETFITS population. However, KLD and ULF dieters made up less than 10% of our study population, which likely reflects differences in personality, experience, and other socioeconomic factors known to affect the response to anchoring (55, 56). Second, the drastic reduction of dietary fat on an ultra low-fat diet may lead to a compensatory increase in the consumption of refined grains that persist even when people increase their fat intake to moderate levels after an initial ULF phase. In contrast, anchoring patients to a ketogenic diet in the first phase of a low-carb diet may lead to greater long-term reductions in added sugars and refined grains. Third, those who follow a KLD in the first 3-months of a low-carb intervention may experience a transient increase in LDL-C with sustained improvements in TG and HDL that persist even when people increase their carbohydrate intakes to moderate non-ketogenic levels of ~80 g/day after an initial KLD phase. While both dietary approaches appear to reduce cardiometabolic risk factors, further research is needed to compare their effects to a Mediterranean Diet in an outcome study with cardiac endpoints and total mortality.

## Data availability statement

The original contributions presented in the study are included in the article/Supplementary material, further inquiries can be directed to the corresponding author.

## Ethics statement

The studies involving human participants were reviewed and approved by Stanford University Human Subjects Committee (Institutional Review Board). The patients/participants provided their written informed consent to participate in this study.

## Author contributions

LA, JR, and CG designed research. JR analyzed data. LA, ML, JR, and CG wrote paper. All authors contributed to the article and approved the submitted version.

## Funding

LA was supported by the European Union’s Horizon 2020 Research and Innovation Program under grant agreement no. 701944. CG was supported by the National Institute of Diabetes and Digestive and Kidney Diseases NIH 1R01DK091831, the Nutrition Science Initiative (NuSI), NIH 1 K12 GM088033, the National Heart, Lung, and Blood Institute NIH T32HL007034 and the Stanford Clinical and Translational Science Award (CTSA) to Spectrum NIH UL1 TR001085.

## Conflict of interest

The authors declare that the research was conducted in the absence of any commercial or financial relationships that could be construed as a potential conflict of interest.

## Publisher’s note

All claims expressed in this article are solely those of the authors and do not necessarily represent those of their affiliated organizations, or those of the publisher, the editors and the reviewers. Any product that may be evaluated in this article, or claim that may be made by its manufacturer, is not guaranteed or endorsed by the publisher.
