# Corrigendum: Weight, insulin resistance, blood lipids, and diet quality changes associated with ketogenic and ultra low-fat dietary patterns: a secondary analysis of the DIETFITS randomized clinical trial

**DOI:** 10.3389/fnut.2023.1275498

**Published:** 2023-10-09

**Authors:** Lucia Aronica, Matthew J. Landry, Joseph Rigdon, Christopher D. Gardner

**Affiliations:** ^1^Stanford Prevention Research Center, Stanford University School of Medicine, Stanford, CA, United States; ^2^Department of Biostatistics and Data Science, Wake Forest University School of Medicine, Quantitative Sciences Unit, Stanford, CA, United States

**Keywords:** ketogenic diet, ultra low-fat diet, low carbohydrate, low fat, weight loss, triglycerides/HDL ratio, insulin resistance, refined grains

In the published article, there was an error in Figure 1 as published. The KLD and ULF bars were switched, but the numerical values within them, as well as other information presented in the figure and caption, were accurate.

The corrected Figure 1 and its caption appear below.

**Figure 1:**
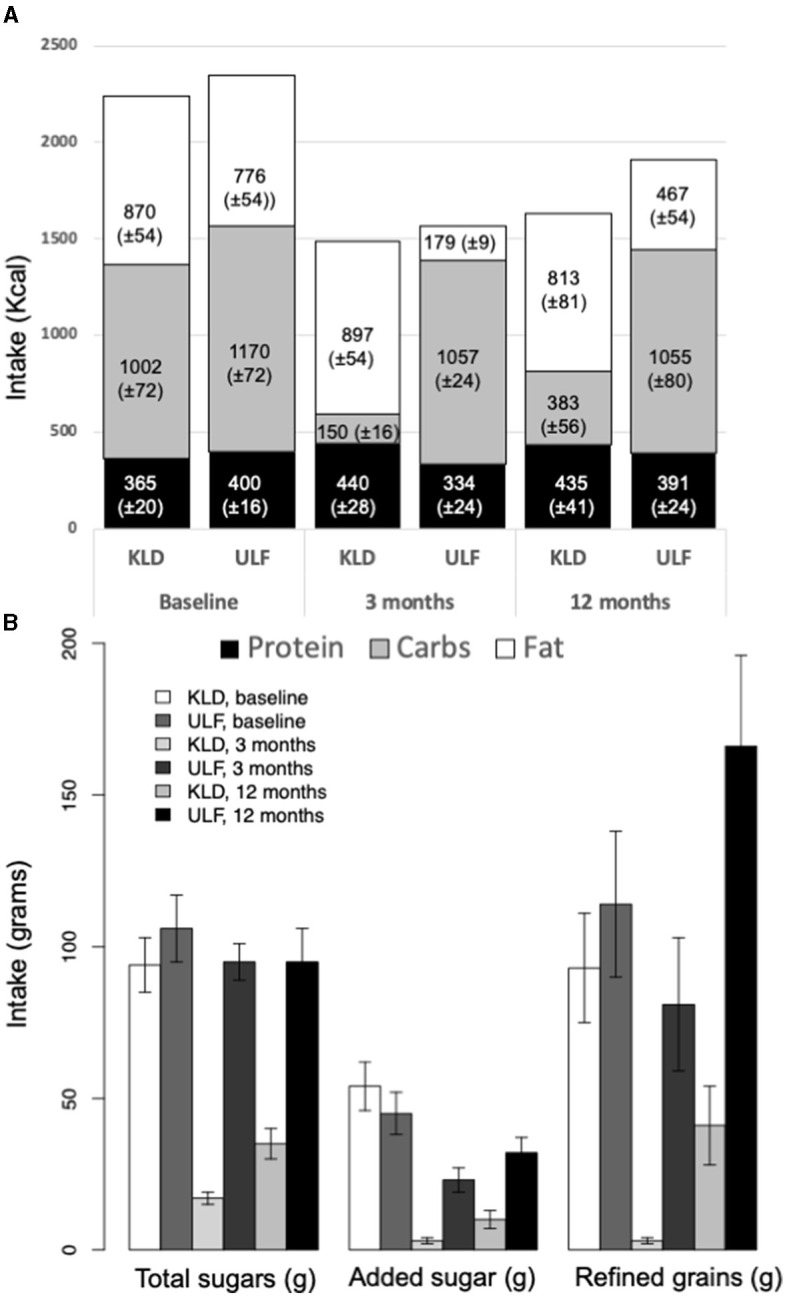
Macronutrient, added sugar, and refined grains intake for KLD and ULF. **(A)**: Mean intake (Kcal/day; ± standard error of mean) of protein (black), carbohydrates (gray), and fat (white) for KLD and ULF at baseline, 3 months, and 12 months. **(B)**: Mean intake (grams/day; ± standard error of mean) of total sugar, added sugars, and refined grains for KLD and ULF at baseline, 3 months, and 12 months. *p*-values for null hypothesis that nutrition variables are equivalent between diets at a given timepoint; from a linear mixed effects model including fixed effects for time, diet, and time^*^diet interaction, and a random effect for study participant.

The authors apologize for this error and state that this does not change the scientific conclusions of the article in any way. The original article has been updated.

