# Supplementary figures and images for: Weight, insulin resistance, blood lipids, and diet quality changes associated with ketogenic and ultra low-fat dietary patterns: a secondary analysis of the DIETFITS randomized clinical trial
Source: Front Nutr. 2023 Jul 12;10:1220020. doi: 10.3389/fnut.2023.1220020 (PMC10369076; doi:10.3389/fnut.2023.1220020)

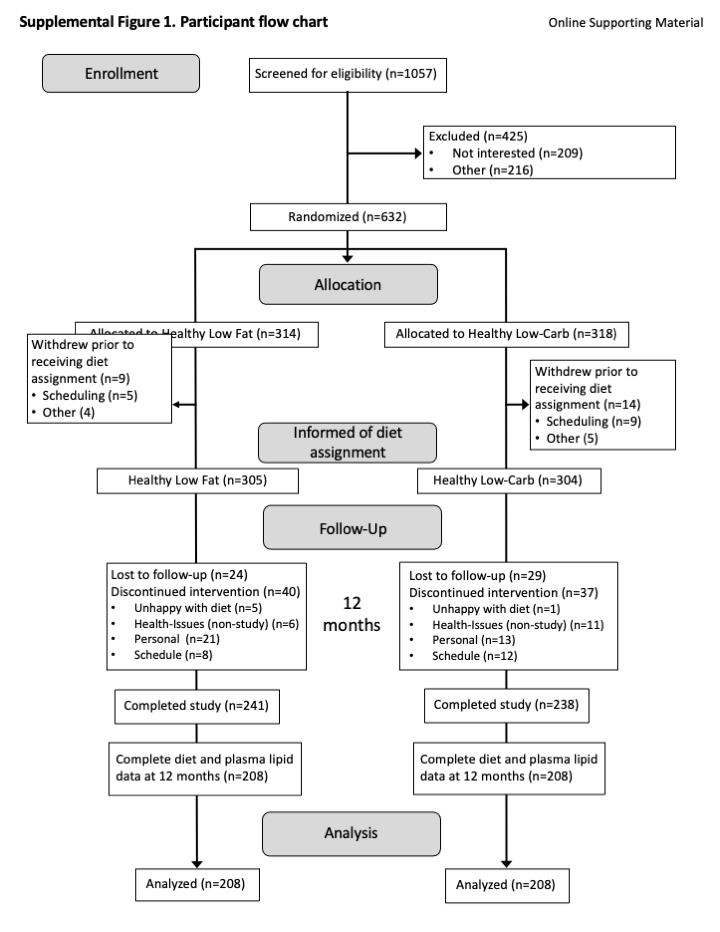

Supplement: Supplementary file 1 [file Image_1.TIFF]
